# Computational Prediction of Drug Solubility in Fasted Simulated and Aspirated Human Intestinal Fluid

**DOI:** 10.1007/s11095-014-1487-z

**Published:** 2014-09-04

**Authors:** Jonas H. Fagerberg, Eva Karlsson, Johan Ulander, Gunilla Hanisch, Christel A. S. Bergström

**Affiliations:** 1 Department of Pharmacy, Uppsala University, Biomedical Centre, P.O. Box 580, SE-751 23 Uppsala, Sweden; 2 Pharmaceutical Development, AstraZeneca R&D, Pepparedsleden 1, SE-431 83 Mölndal, Sweden; 3 Cardiovascular & Metabolic Diseases, AstraZeneca R&D, Pepparedsleden 1, SE-431 83 Mölndal, Sweden

**Keywords:** biorelevant solubility, human intestinal fluid, simulated intestinal fluid, in silico, prediction

## Abstract

**Purpose:**

To develop predictive models of apparent solubility (S_app_) of lipophilic drugs in fasted state simulated intestinal fluid (FaSSIF) and aspirated human intestinal fluid (HIF).

**Methods:**

Measured S_app_ values in FaSSIF, HIF and phosphate buffer pH 6.5 (PhB_pH6.5_) for 86 lipophilic drugs were compiled and divided into training (Tr) and test (Te) sets. Projection to latent structure (PLS) models were developed through variable selection of calculated molecular descriptors. Experimentally determined properties were included to investigate their contribution to the predictions.

**Results:**

Modest relationships between S_app_ in PhB_pH6.5_ and FaSSIF (R^2^ = 0.61) or HIF (R^2^ = 0.62) were found. As expected, there was a stronger correlation obtained between FaSSIF and HIF (R^2^ = 0.78). Computational models were developed using calculated descriptors alone (FaSSIF, R^2^ = 0.69 and RMSE_te_ of 0.77; HIF, R^2^ = 0.84 and RMSE_te_ of 0.81). Accuracy improved when solubility in PhB_pH6.5_ was added as a descriptor (FaSSIF, R^2^ = 0.76 and RMSE_Te_ of 0.65; HIF, R^2^ = 0.86 and RMSE_Te_ of 0.69), whereas no improvement was seen when melting point (Tm) or logD_pH 6.5_ were included in the models.

**Conclusion:**

Computational models were developed, that reliably predicted S_app_ of lipophilic compounds in intestinal fluid, from molecular structures alone. If experimentally determined pH-dependent solubility values were available, this further improved the accuracy of the predictions.

## Introduction

Modern drug discovery programs using high throughput screening and combinatorial chemistry continue to favor the selection of large and lipophilic new chemical entities (NCE). This is in spite of their poor aqueous solubility [1–4], the increased awareness of related problems and the multitude of mnemonic rules for avoiding these compounds with low or variable absorption and pharmacokinetics [5–7]. Solubility in intestinal fluids is a key property for estimating absorption of oral drugs and in this context aspirated human intestinal fluid (HIF) has been described as the gold standard medium for these estimations [8,9]. However, there are disadvantages associated with its use. Ethical concerns about HIF sampling regulate the availability of the fluid. The amount available to the scientific community is therefore sparse and expensive. Other concerns are its low buffer capacity and batch variation in pH and bile content. These variations are due to differences in aspiration protocols and individual differences between volunteers [9,10], although differences due to the latter can be somewhat alleviated by pooling samples. Alternatives to HIF for dissolution testing and solubility measurements became available when Dressman and colleagues introduced fasted state simulated intestinal fluid (FaSSIF) in 1998 [11]. Other biorelevant dissolution media (BDM) have been developed since then, some to closer mimic the intestinal milieu [12,13] and others for ease of preparation or lower expense [14,15]. Nonetheless the use of FaSSIF continues to be widespread and a large number of compounds and formulations have been evaluated in them. The medium contains taurocholate and lecithin that form mixed lipid aggregates in the form of vesicles which are colloidal structures known to efficiently solubilize drug molecules. The extent of solubilization is dependent on lipid concentration and substance-specific properties such as size, charge, flexibility, and lipophilicity. Drugs with a partition coefficient between octanol and water (logP) greater than 3 have considerably higher apparent solubility (S_app_; the total concentration of drug dissolved in the lipid-containing dissolution medium) in BDM than in water or buffers [16–19]. Solubilization in lipid aggregates and molecular ionization as a response to the pH of the fluid can increase the S_app_ in BDM of compounds several orders of magnitude compared to that observed in water.

Solubility measurements are time consuming. More importantly, the substances of interest must be synthesized before their solubility can be evaluated. Computational predictions of solubility, on the other hand, are rapid and can be performed on large compound libraries without synthesis of the substances. This provides the medicinal chemists with solubility profiles on which they can make better informed decisions, and the costs associated with the pharmaceutical profiling cycle are reduced because of decreased demand for expensive simulated or aspirated intestinal fluid. Solubility predictions in BDM, or even better HIF, are therefore highly warranted.

Numerous models for the prediction of intrinsic aqueous solubility (S_0_), *i.e.*, the solubility of the neutral compound, have been developed [20]. One of the most renowned is the general solubility equation (GSE) [21] and derivatives thereof [22, 23]. These are based on logP, which typically can be computationally predicted with an RMSE of around one log_10_ unit, and experimentally determined melting temperature (Tm). The latter is sometimes replaced with other properties more amenable to trivial calculations or prediction such as MW [24]. Aqueous solubility can also be successfully predicted using calculated molecular descriptors, see *e.g.* [25,26],. However, in the gastrointestinal tract, pH values range from ~2.5 in the stomach to ~6.9 in the jejunum [10]. This pH-gradient greatly impacts the ionization of protolytic compounds and hence, the observed S_app_ is dependent on the extent of ionization of a particular molecule. The pH-dependent solubility can be calculated from S_0_ and the dissociation constant (pKa) with the Henderson-Hasselbalch equation [27]. However, the accuracy of these estimations varies considerably because the Henderson-Hasselbalch equation does not take into account aggregation or common ion/salt effects [28]. The complexity increases even more when solubility is measured in BDM since the apparent solubility in these media is a result of ionization, aggregation and solubilization. We have previously attempted to predict S_app_ in biorelevant media using a small dataset [16,17]. A predictive artificial neural network (ANN) model for FaSSIF solubility is also available in the commercial software ADMET Predictor from Simulations Plus. However, no transparent models for prediction of FaSSIF S_app_ have been developed using publicly available solubility data for drugs, nor have any predictive models of HIF S_app_ been proposed. Here, we report an open database applicable for solubility modeling in FaSSIF and HIF. This database has been used to develop transparent and reliable models for the prediction of solubility in FaSSIF and HIF with the aim of revealing molecular features that drive solubilization in these fluids.

## METHODS

### Datasets

S_app_ values for 86 drugs in FaSSIF (3 mM taurocholate, 0.75 mM lecithin in PhB_pH6.5_ [11]) were extracted from in-house databases [8,16,17,29] and literature sources [30–38] (Table I). To reduce experimental variability in the dataset the main part of the compounds was obtained from our in-house databases in which solubility measurements taking use of shake-flask or the μDISS Profiler are reported. Only compounds with a calculated logP greater than 2 were included since it is assumed that there is significant solubilization of highly lipophilic compounds in the mixed lipid aggregates present in FaSSIF [16–19]. Hence, we argue that for compounds with log *P* < 2, *in silico* models predicting solubility in pH-adjusted water/simple buffer are also predictive of their solubility in intestinal fluid (Fig. S1). The FaSSIF S_app_ values were supplemented with the corresponding S_app_ values in PhB_pH6.5_ and the Tm, when available, for the free base or free acid (*i.e.*, not salts).

**Table I:** Physicochemical properties and S_app_ of the investigated compounds^a^

Compound	Set	Mw (Da)	logD _pH6.5_	Polar Surface Area (Å^2^)	Rotable bonds (count)	log S_app_ PhB _pH6.5_ (M)	log S_app_ FaSSIF (M)	log S_app_ HIF (M)	Tm (°C)
Albendazole	Te	265.3	3.2	79.7	6	−5.49	−5.14	−4.45	178.1
Amiodarone	Tr	645.3	5.2	193.0	9	−7.30	−3.26	−3.23	159.0
Amitriptyline	Tr	277.4	2.4	2.2	3	−2.49	−2.50		196.5
Amprenavir	Tr	505.6	1.7	138.0	13	−3.47	−3.65	−3.74	72.0
Aprepitant	Te	534.4	4.4	325.0	8	−6.16	−4.37	−4.61	252.0
Astemizole	Tr	458.6	4.4	57.6	8	−4.38	−3.67	−4.68	174.4
Atovaquone	Tr	366.9	3.7	155.0	2	−5.93	−5.29	−6.06	224.0
Azelnidipine	Te	582.7	6.0	174.0	8		−3.97		122.0
Bicalutamide	Tr	430.4	2.6	251.0	7		−4.65		273.0
Bromazepam	Tr	316.2	1.8	144.0	0		−3.42		243.0
Bromocriptine	Tr	654.6	3.6	167.0	6		−4.05		215.0
Carbamazepine	Tr	236.3	2.6	73.5	1	−3.27	−3.00	−2.92	191.0
Carvedilol	Te	406.5	2.4	70.9	10	−3.95	−3.86	−4.05	114.1
Celecoxib	Tr	381.4	3.5	222.0	2		−4.29		158.0
Cilostazole	Te	369.5	2.8	139.0	7	−4.77	−4.76		159.0
Cinnarizine	Tr	368.5	4.3	2.6	5	−5.42	−4.44	−4.28	119.0
Cisapride	Tr	466.0	2.6	182.0	9	−5.27	−4.86		110.0
Clotrimazole	Tr	344.9	5.2	50.4	4	−5.17	−5.00	−4.13	142.0
Cyclosporine	Te	1202.6	3.0	179.0	15	−5.80	−5.32	−5.54	150.0
Danazol	Tr	337.5	3.6	68.8	1	−5.75	−4.60	−4.84	227.0
Diazepam	Te	284.8	2.9	105.0	0	−3.91	−3.64	−3.28	131.6
Diclofenac	Tr	296.2	2.2	169.0	4	−2.78	−2.59	−2.52	158.0
Diethylstilbestrol	Tr	268.4	4.8	80.2	2	−4.31	−3.83	−3.85	171.0
Digoxin	Te	781.0	1.4	241.0	7	−4.69	−4.66		249.0
Dipyridamole	Te	504.6	1.8	103	12	−4.9	−4.64	−4.24	163.0
Disopyramide	Te	339.5	−0.2	58.7	8	−3.24	−3.03		96.4
Efavirenz	Te	315.7	3.9	214.0	3		−3.41		139.0
Felodipine	Tr	384.3	4.8	136.0	4	−5.51	−3.85	−4.44	143.0
Fenofibrate	Te	360.8	5.3	138.0	5	−6.26	−4.58	−4.26	79.0
Flufenamic acid	Tr	281.2	2.6	167.0	3	−2.75	−2.48	−2.82	133.5
Fluoxetine	Tr	309.3	1.6	109.0	7		−2.35		132.8
Gefitinib	Te	446.9	3.8	147.0	8	−5.04	−3.72	−3.72	119.0
Glibenclamide	Tr	494.0	3.9	203.0	10	−5.04	−5.02	−4.12	173.6
Griseofulvin	Tr	352.8	2.5	135.0	3	−4.38	−4.18	−4.32	219.0
Halofantrine	Te	500.4	6.0	252.0	11		−4.07		77.0
Haloperidol	Tr	375.9	2.0	160.0	5	−3.68	−3.53		151.0
Ibuprofen	Tr	206.3	1.8	73.8	4		−2.17	−2.02	76.0
Indinavir	Tr	613.8	3.0	72.5	14	−3.90	−4.31	−4.16	167.5
Indomethacin	Te	357.8	1.5	185.0	3	−3.21	−2.91	−2.46	159.8
Indoprofen	Te	281.3	0.7	102.0	3	−2.98	−2.66		211.4
Irbesartan	Tr	428.5	4.0	108.0	5	−3.62	−3.58	−3.54	180.5
Isotretinoin	Te	300.4	4.4	74.7	0		−3.76		174.0
Ivermectin	Te	875.1	4.7	112.0	8	−6.10	−3.86		150.0
Ketoconazole	Tr	531.4	3.9	181.0	8	−4.56	−3.50	−3.98	146.0
Lansoprazole	Tr	369.4	1.8	161.0	6	−4.17	−3.97		178.0
Loperamide	Tr	477.1	3.9	112.0	8	−4.04	−3.67		130.0
Lopinavir	Te	628.8	4.2	67.0	17	−5.76	−4.04	−4.67	124.0
Lorazepam	Tr	321.2	2.6	213.0	0	−3.44	−2.93		167.0
Loviride	Tr	351.2	3.2	177.0	4	−5.55	−4.92	−3.71	286.8
Naproxen	Tr	230.3	1.3	84.5	3	−3.00	−2.67	−2.05	155.6
Nefazodone	Tr	470.0	3.9	110.0	10		−3.27		83.5
Nelfinavir	Te	567.8	4.7	83.8	11	−6.16	−3.68	−4.01	349.8
Nevirapine	Tr	266.3	1.7	55.9	1	−3.51	−3.14		196.1
Nimesulide	Tr	308.3	2.3	164.0	4	−4.13	−3.93	−3.56	144.0
Nitrendipine	Tr	360.4	3.5	126.0	4	−4.95	−4.35	−4.95	157.7
Omeprazole	Tr	345.4	1.9	76.9	5	−3.28	−3.10	−3.03	156.0
Panadiplon	Tr	335.4	2.5	57.5	2	−3.64	−3.60		169.0
Phenazopyridine	Tr	213.2	2.8	115.0	0	−3.08	−2.67		139.0
Phenytoin	Tr	252.3	2.2	113.0	2	−3.81	−3.77		295.6
Pranlukast	Tr	481.5	3.3	177.0	8	−5.17	−3.75		236.0
Praziquantel	Te	312.4	2.2	55.0	2	−3.17	−3.08		139.0
Probenecid	Te	285.4	0.0	132.0	6	−2.34	−2.24	−2.59	198.9
Probucol	Te	516.9	10.0	14.9	8	−8.94	−5.18	−5.75	126.0
Progesterone	Tr	314.5	3.8	63.1	1	−4.45	−4.09	−4.08	128.0
Quinidine	Tr	324.4	1.5	39.4	4	−2.19	−2.16	−2.70	174.0
Rifampicin	Te	823.0	3.0	158.0	4	−2.92	−2.61	−2.14	183.0
Riluzole	Tr	234.2	2.3	159.0	2		−2.58		119.0
Rimonabant	Tr	463.8	5.3	227.0	2	−6.39	−4.62	−4.93	154.7
Ritonavir	Tr	721.0	4.6	77.9	22	−5.27	−5.07	−4.32	120.0
Rofecoxib	Tr	314.4	2.7	119.0	1	−4.61	−4.53		207.0
Salsalate	Tr	258.2	0.8	119.0	2		−2.21		147.0
Saquinavir	Te	670.9	4.1	152.0	15	−3.93	−3.57	−4.24	349.8
Sertraline	Tr	306.2	2.8	135.0	2		−3.10		219.0
Spironolactone	Te	416.6	3.0	111.0	2		−4.21		134.5
Sulfasalazine	Tr	398.4	0.3	188.0	5	−3.49	−3.34	−2.86	255.0
Tamoxifen	Tr	371.5	4.8	8.6	5	−4.80	−3.38	−3.77	97.8
Tamsulosin	Te	408.5	1.1	121.0	11		−2.45		226.0
Telmisartan	Tr	514.6	4.5	79.8	4		−5.37		269.0
Terfenadine	Tr	471.7	3.5	46.2	9	−4.62	−3.74		149.6
Tipranavir	Tr	602.7	6.0	189.0	12	−5.19	−4.47	−4.00	86.0
Tolectin	Te	257.3	0.1	99.5	2		−2.14		156.0
Tolfenamic acid	Tr	261.7	2.9	129.0	2	−3.98	−3.62		213.0
Troglitazone	Te	441.6	4.9	143.0	5		−4.96		184.0
Warfarin	Tr	308.3	3.1	70.8	4	−3.19	−2.94	−2.99	161.0
Voriconazole	Tr	349.3	2.1	146.0	5		−2.72		127.0
Zafirlukast	Te	575.7	4.1	123.0	10		−5.44		139.0
Min		206.3	−0.2	2.2	0	−8.94	−5.44	−6.06	72.0
Max		1202.6	10.0	325.0	22	−2.19	−2.14	−2.02	349.8
Median		369.0	3.0	122.0	5	−4.38	−3.73	−4.00	158.0

^a^Set indicates training (Tr) or test (Te) set classification in the FaSSIF models. All compounds listed were used as the training set in the HIF model. MW represents molecular wheight in Daltons. logD_pH6.5_ is the logarithm of octanol/water partitioning at pH 6.5, as predicted from the ADMET predictor (Simulations Plus, CA). This software was also used to calculate polar surface area and number of rotatable bonds. Apparent solubility in PhB_ph6.5_, FaSSIF and HIF is expressed as the logarithm of molar equilibrium concentration

This same approach was used in the selection of a dataset for which solubility in aspirated HIF was available (Table I). An additional criterion for this dataset was that corresponding solubility measurements were available in FaSSIF and that the same protocol had been used for solubility measurements in FaSSIF and HIF. The final HIF dataset consisted of 48 compounds. In addition to the FaSSIF and HIF literature datasets, a discovery dataset of 26 AstraZeneca proprietary compounds for which the S_app_ in FaSSIF, HIF and PhB_pH6.5_ were available was used as an external validation dataset for the developed models.

### Calculation of Molecular Descriptors

Molecular structures for the compounds were obtained as SMILES strings (Table SI) and converted to energy-minimized, three-dimensional structures with added implicit hydrogens using Corina 3.49 (Molecular Networks, Germany). Molecular descriptors from the resulting structures were generated using DragonX 6.0.16 (Talete, Italy). The descriptors were blinded to avoid selection bias, cubic-root transformed, mean-centered and scaled to unit variance. Thereafter, any descriptors not displaying normal distribution were excluded from the model development.

We also used ADMET Predictor 6.5 (Simulations Plus, CA) to predict FaSSIF S_app_ for the compounds and to calculate pKa (Table SI) and pH-dependent lipophilicity at pH 6.5 (logD_pH6.5_), polar surface area (PSA), and number of rotatable bonds, see Table I. The predictions of FaSSIF were used as a comparator for our predictions.

### In Silico Model Development

Partial least squares projection to latent structures (PLS) models were developed with the purpose of predicting S_app_ values in FaSSIF and in HIF. The respective models were developed with Simca-P 13.0.2.0 (Umetrics, Sweden) using a standardized protocol previously implemented by our group [39,40]. The responses were used in the logarithmic form of the solubility in the two different media. For the FaSSIF model, the compounds were randomized into a training (Tr, *n* = 56) and a test (Te, *n* = 30) set. The compounds were sorted by their S_app_ to achieve an even distribution and as wide a predictive range as possible. Every third compound was then assigned to the test set. The structural diversity and the suitability of the selected test set was thereafter tested by a principal component analysis (PCA) extracted from all descriptors. Any training set outliers identified in the PCA (Supporting Information, Fig. S2) or distance–to–the–model–of–X [41] (DmodX) were moved to the corresponding test set to avoid such compounds weighting the training of the models. PCA was also used to ensure that the training and test sets were well distributed in the chemical space. In addition to the literature-derived test set, a discovery dataset of 26 AstraZeneca in-house compounds was used to challenge the model. For the HIF model, these discovery compounds were used as the sole test set while the training set consisted of the literature values. PCA confirmed that the chemical space of this dataset was covered by the training set (Fig. S2).

All but the top 100 descriptors (Table SII) (as defined by variable of importance–to–projection, VIP) were excluded in the first step of model development. The variable selection procedure was thereafter based on the VIP and the loading plots, and monitored by the leave-one-out (using 7 groups), cross-validated R^2^ (Q^2^). The variable selection was performed to remove non-significant descriptors and increase model robustness. If the exclusion of a variable resulted in an equal or improved Q^2^, the variable was permanently eliminated from the model. The variable selection procedure was repeated until no further descriptors could be removed without a resultant lower Q^2^. Only thereafter was the accuracy of the prediction of the test set investigated.

In the second step, the impact of experimental data commonly available during early development was investigated by adding such measured data to the final model obtained after the completion of variable selection. The investigated experimental data were S_app_ in PhB_pH6.5_, Tm, and logD_pH6.5_. Measured PhB_pH6.5_ S_app_ was available for 76% of the compounds (Table I). It was not possible to extract measured logD_pH6.5_ for a large number of the compounds and therefore the calculated logD_pH6.5_ (ADMET Predictor, Simulations Plus, CA) was used for all of them. Whether these properties were beneficial or not for the models was evaluated in the same manner as for the calculated descriptors, see above.

In addition to the models above, a consensus model was established which used the developed FaSSIF PLS model based on calculated descriptors only and the predictions obtained from ADMET Predictor ANN model. No weighting was performed. The consensus model used the average of the predicted logS_app_ from the developed PLS model and the commercial ANN model.

## Results

### Physicochemical Properties and Apparent Solubility

The datasets used were structurally diverse. The FaSSIF modelling dataset (Table I) ranged in size from 206.3 to 1202.6 Da with a median mass of 369; hydrogen bond capacity in the form of PSA ranged from 2.2 to 325 Å^2^ with a median of 122 Å^2^; and molecular flexibility (described by the number of rotatable bonds count) ranged from 0 to 22, with a median of 5. The dataset was selected to focus on lipophilic compounds and therefore all compounds had a calculated AlogP-value >2 (obtained from the software DragonX). In spite of this lipophilicity criterion, the predicted pH-dependent lipophilicity logD_6.5_ values from ADMETPredictor ranged over 10 orders of magnitude, from -0.2 to 10.0. The solubility varied 2000- and 10,000-fold in FaSSIF and HIF, respectively (Table I).

**Fig. 1:**
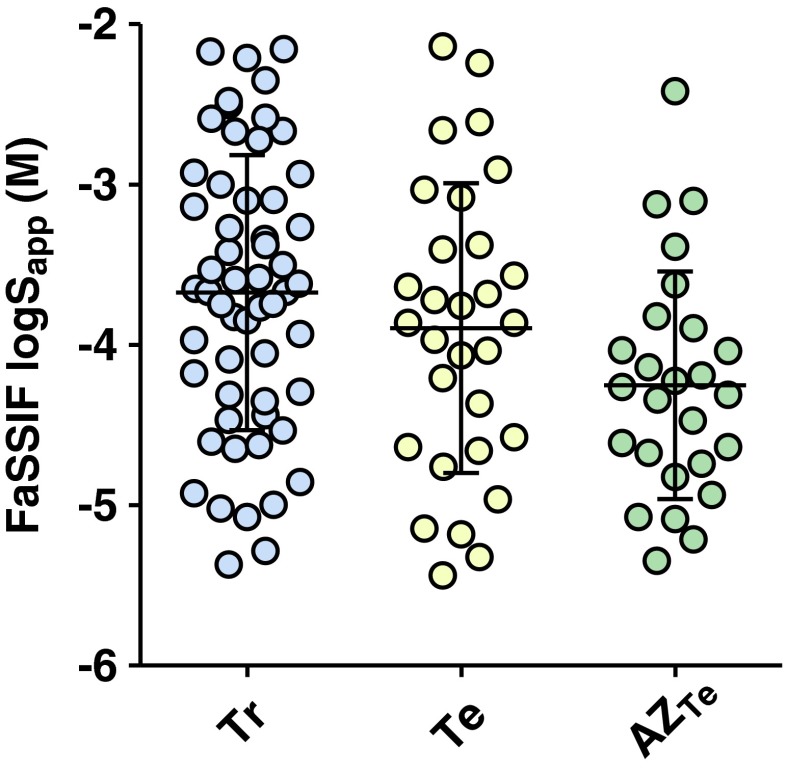
Training- and testset S_app_ ranges for FaSSIF. The literature training (Tr) and test sets (Te) are shown with blue and yellow circles respectively and the discovery test set is denoted with green circles.

The solubility range of the training set was similar to that of the literature test set, whereas the 26 discovery compounds used to challenge the models had a somewhat lower solubility (Fig. 1).

**Fig. 2:**
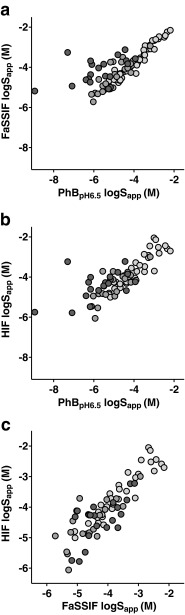
Correlation between HIF, FaSSIF and buffer pH 6.5 Sapp correlations. **a** FaSSIF and PhBpH6.5, **b** HIF and PhBpH6.5, and **c** HIF and FaSSIF. Light gray circles represent compounds with a predicted logD_pH6.5_ below 3. Gray circles denote compounds with logD_pH6.5_ between 3 and 4. Dark gray circles shows compounds with a logD_pH6.5_ above 4.

The solubility values of the dataset revealed modest relationships between measured solubility in PhB_pH6.5_ and FaSSIF or HIF (R^2^:0.61 and R^2^:0.62, respectively, Fig. 2). The PhB_pH6.5_ and FaSSIF S_app_ correlation was considerably weaker for the more lipophilic compounds (logD_pH6.5_ > 4) for which R^2^ decreased to 0.28. However, for compounds with a logD_pH6.5_ < 3, there was a strong correlation (R^2^: 0.82) The correlation between FaSSIF S_app_ and HIF S_app_ was also strong (R^2^: 0.78) but unaffected by lipophilicity (Fig. 2c). Under- or over-prediction of the solubility was not related to melting point or lipophilicity.

### Prediction of Intestinal Solubility

**Fig. 3:**
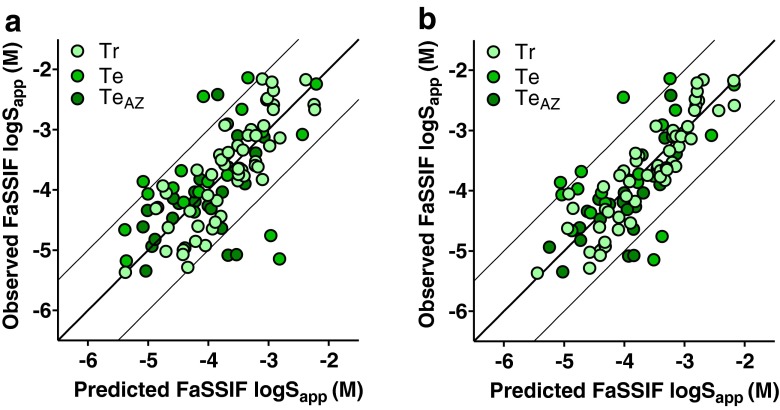
Prediction results for solubility in FaSSIF. **a** FaSSIF model based on seven calculated descriptors and **b** the same but including measured buffer solubility. Light green circles represent the training set while green and dark green circles denote the literature test set and discovery test set, respectively.

**Fig. 4:**
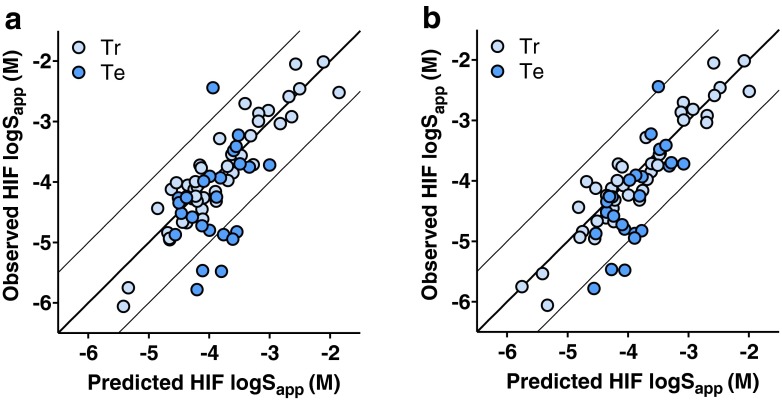
Prediction results for solubility in HIF. **a** HIF model based on nine calculated descriptors and **b** the same but including measured buffer solubility. Light blue circles show the literature training set while blue circles represent the discovery compound test set.

The developed PLS models are summarized in Table II. The FaSSIF model (Fig. 3a) required seven calculated descriptors to produce two principal components resulting in R^2^ of 0.69, Q^2^ of 0.64 and an RMSE_tr_ of 0.48 log_10_ units. The HIF model (Fig. 4a), based on nine descriptors, had a higher R^2^ of 0.84 (Q^2^ of 0.78) and a lower RMSE_tr_ (0.34). The inclusion of experimentally determined S_app_ in PhB_pH6.5_ strengthened the predictive power of both models to R^2^ of 0.76 (FaSSIF) and 0.86 for HIF (Figs. 3b and 4b, respectively), and it reduced the RMSE of the test sets (Table II). Inclusion of Tm and logD_pH6.5_ did not improve the developed models. Further, these properties were unable to identify over- or under-predicted compounds or any clusters.

**Table II:** Model Summary.

Model	Descriptors	R2	Q2	RMSE_Tr_	RMSE_Te_	n in Te
FaSSIF	7 calculated	0.69	0.64	0.48	0.77	49
Tr n:56	+ S_app_ in PhB_pH 6.5_	0.76	0.70	0.39	0.65	47
HIF	9 calculated	0.84	0.78	0.34	0.80	26
Tr n:43	+ S_app_ in PhB_pH 6.5_	0.86	0.79	0.32	0.68	26

**Fig. 5:**
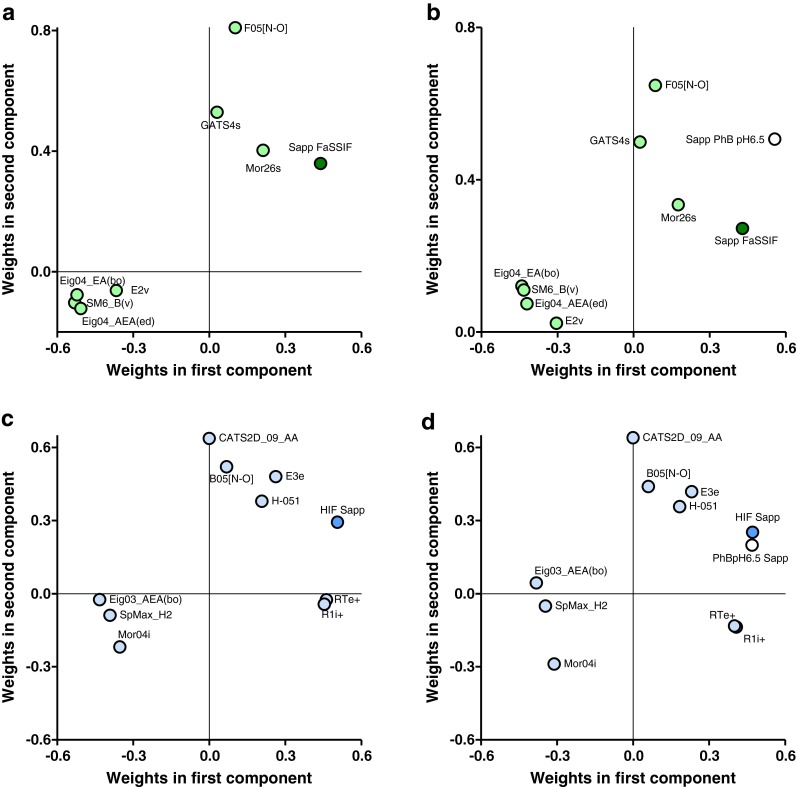
Loading plots for the FaSSIF and HIF models. **a** FaSSIF model loading plot and **b** with measured PhBph6.5 Sapp as additional descriptor. **c** HIF model loading plot and **d** experimentally determined PhBph6.5 Sapp as a_descriptor.

All descriptors remaining after the variable selection were significant in either both or the last component. For the FaSSIF model these include: i) eigenvalues weighted by bond order (Eig04_EA(bo)) or edge degree (Eig04_AEA(ed)); ii) a spectral moment of order 6 from Burden matrix weighted by van der Waals volume; and iii) a second-component accessibility directional WHIM index weighted by van der Waals volume; all these descriptors negatively influenced the solubility. These descriptors are related to some extent to molecular size. Geary autocorrelation (GATS4s) and Morse signal 26(Mor26s), both weighted by intrinsic state and the frequency of N – O at a topological distance of 5 (F05[N-O]) correlated with a high S_app_ in FaSSIF (Fig. 5a and b).

The HIF model descriptors included an eigenvalue weighted by bond order (Eig03_AEA(bo)), Morse signal 4 weighted by ionization potential (Mor04i) and an eigenvalue from reciprocal squared distance matrix (SpMax_H2); these were found to limit the S_app_. The HIF S_app_ was further positively influenced by the following descriptors:, R maximal index (RTe+) and a WHIM index (E3e) both weighted by Sanderson electronegativity, R maximal autocorrelation weighted by ionization potential (R1i+), the presence of N – O at topological distance 5(B05[N-O]), CATS2D acceptor-acceptor at lag 09 (CATS2D_09_AA) and hydrogen attached to alpha carbons (H-051) (Fig. 5c and d).

The model using calculated descriptors over-predicted the FaSSIF S_app_ of albendazole and cilostazole by one logarithmic unit or more. On the other hand, ivermectin, tamsulosin, and tolectin were all under-predicted by one log_10_ unit or more. The inclusion of measured S_app_ in PhB_pH6.5_ as a descriptor improved the predictions for cilostazole and albendazole and reduced their residual values by 0.41 and 0.69 log_10_ units respectively.

**Fig. 6:**
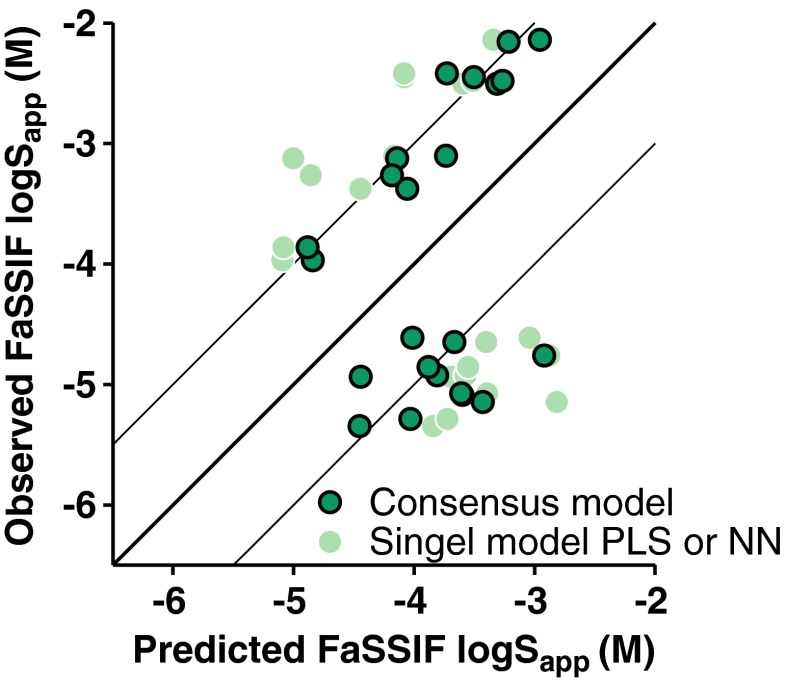
Consensus model for compounds with residuals over 1 log10 unit in any model based on calculated descriptors. Light green circles without outline show the worst prediction from either the ANN or PLS model. Outlined dark turquoise circles represent the consensus model prediction.

The consensus model based on the calculations obtained from our FaSSIF model and the ADMET Predictor results exhibited a lower RMSE_te_ (0.70) compared to each of the models separately (Table III). The PLS model was however more accurate compared to the consensus model in the prediction of the solubility of the discovery test set. Importantly, predictions of outliers resulting from each of the PLS and ANN models were greatly improved by consensus modelling (Fig. 6).

**Table III:** Consensus model performance.^a^

Test Set	FaSSIF_PLS_	FaSSIF_ANN_	FASSIF_Consensus_
Combined_Te_	0.77	0.82	0.70
Literature_Te_	0.89	0.73	0.71
AZ_Te_	0.62	0.91	0.69

^a^RMSEs for the different test sets of the developed PLS model, the commercial ANN model, and the resulting average consensus model

## Discussion

Intestinal solubility, together with permeability over the intestinal wall, are the two most important drug properties determining absorption after oral intake. Solubility measurements in HIF will continue to be important in understanding intestinal solubility, but the medium is expensive and subject to batch variations. Therefore, a number of BDMs have been developed as robust and reproducible surrogates. The strong correlation between FaSSIF and HIF S_app_ found herein confirms those reported previously [8, 9] and further supports the use of *in vitro* experiments in BDMs, such as FaSSIF, for the prediction of intestinal solubility.

The aim of this study was to develop predictive models for S_app_ in FaSSIF and HIF using calculated descriptors alone or in conjunction with experimental data likely to be available in early drug discovery or development stages. The descriptors included in the final FaSSIF model can be used to interpret molecular properties of importance for solubility in FaSSIF. The descriptors reveal that larger structures are solubilized to a lesser extent than the smaller ones. Most likely this is as a result of the increased cavity that needs to be formed in the water as well as the increased molecular surface area exposed to the water. In addition, aromatic structures were revealed to be less hydrated than aliphatic ones. We speculate that this could be because of their stronger crystal lattices, due to the stronger van der Waals interactions formed by the dense packing. Further, when in the water, rigid aromatic structures have a larger molecular surface area exposed to the solvent than flexible aliphatic chains that can change conformation to shield the carbon skeleton from water molecules. The descriptors also identify the importance of hydrogen bond donor and acceptors for the hydration of the molecule. Although the descriptors in the HIF model differ to some extent from those of the FaSSIF model, they too reflect similar properties.

During the model development we tested three empirical or semi-empirical descriptors. The addition of experimentally determined S_app_ in PhB_pH6.5_ as a descriptor improved both developed PLS models considerably. This solvent can be regarded as a blank FaSSIF because it is a phosphate buffer (pH 6.5) that does not contain any mixed lipid aggregates. Ionizable compounds are therefore charged to the same extent in both media and information on this effect facilitates the prediction. The relationship between PhB_pH6.5_ and FaSSIF S_app_ was modest and weak for the lipophilic compounds. It is therefore interesting to note that such a divergence was not seen in the PLS predictions regardless of whether or not PhB_pH6.5_ was included as an experimentally determined descriptor. Tm and lipophilicity expressed as logP or logD_pH6.5_ are common inputs for aqueous solubility predictions, and were therefore tested herein. However, neither logD_pH6.5_ nor Tm improved the developed models. Intestinal fluids contain lipid aggregates that may solubilize lipophilic drug molecules and it is well-known that logP is not a good descriptor of solubility in lipids, see *e.g.* [40],. The reduced solubility of the bulk water seen in intestinal fluids with higher logD_pH6.5_ is to some extent compensated by partitioning to and/or solubilization in the aggregates. To elucidate if Tm or logD_pH6.5_ were described to some extent by the selected calculated descriptors these properties were used as responses in the models. We found no indication of correlation between the selected descriptors and lipophilicity or solid state properties of the compounds (R^2^ < 0.45). An interesting aspect of the increased accuracy of the predictions when S_app_ in PhB_6.5_ is included as descriptor is that the influence of the solid state on the S_app_ is diminished. Hence, the PhB_6.5_ contributes to better predictions at two levels; the hydration is better described as a result of the correct description of the pH-dependent solubility and the impact of the dissociation of molecules from the crystal lattice is embedded in this solubility input variable.

It was possible to further improve the FaSSIF predictions by performing consensus modeling based on the combination of two different computational models that used only calculated descriptors. The combination of the (ANN) FaSSIF model results (obtained from ADMET Predictor) with the PLS predictions increased the predictive power, as identified from the lowered RMSE_te_ (Table III). Three test sets were evaluated: all test compounds (*n* = 75), a literature-derived test set (*n* = 49), and a discovery test set of AstraZeneca proprietary compounds (*n* = 26). For all three, the consensus model performed better than the worst-performing computational model. Most importantly, the consensus model increased the robustness and reduced the number of outliers (Fig. 6). Indeed, for the 23 compounds that were 10 to 320-fold over- or under-predicted by either of the two models, the employment of the consensus model reduced the RMSE to <1 log unit for 12 of them. Of these 23 compounds, 14 were significantly falsely predicted by the ANN, 5 by the PLS, and 4 by both. Since it is difficult to deem beforehand which one of several models will be the most accurate predictor for any new compound or compound series, it is advisable to employ consensus modelling based on two or more models.

There are a number of hurdles to allow increased accuracy in solubility predictions in BDM. The models developed herein that are based on calculated descriptors alone are reliable and statistically strong, but the observed residual errors do imply that the predictions could be up to tenfold off in either direction. This is not uncommon for predictions of solubility in aqueous media and the developed models are certainly accurate enough for guiding decision-making in drug discovery and development. To further improve the computational predictions of solubility in media containing mixed lipid aggregates molecular dynamics (MD) simulation is a promising tool to study solubilization interactions [42,43]. MD simulation also has the potential to predict self-aggregation and the tendency to form mixed micelle aggregates. Food effects on bioavailability is another issue related to BDM and larger datasets need to be studied for solubility in fed state BDM to obtain information on molecular features of drug molecules that are significantly affected by the increased lipid content in the fed state.

## Conclusions

Measurements of solubility in physiologically relevant media such as FaSSIF and HIF are costly and in part restricted by the limited access to intestinal fluids aspirated from donors. Herein we present computational approaches to instead predict intestinal solubility taking use of calculated molecular descriptors to allow this property to be estimated already before compound synthesis. It was found that the S_app_ of lipophilic compounds in FaSSIF and in HIF was possible to predict by this approach. The most accurate predictions were obtained when a consensus modeling approach was used, which reduced the number of outliers obtained from predictions based on a single computational model. Further, we have examined experimental parameters within reach during early drug development and identified pH-dependent solubility as a descriptor that further increases the accuracy of the predictions.
